# The human ZC3H3 and RBM26/27 proteins are critical for PAXT-mediated nuclear RNA decay

**DOI:** 10.1093/nar/gkz1238

**Published:** 2020-01-17

**Authors:** Toomas Silla, Manfred Schmid, Yuhui Dou, William Garland, Miha Milek, Koshi Imami, Dennis Johnsen, Patrik Polak, Jens S Andersen, Matthias Selbach, Markus Landthaler, Torben Heick Jensen

**Affiliations:** 1 Department of Molecular Biology and Genetics, Aarhus University, C.F. Møllers Allé 3, 8000 Aarhus C, Denmark; 2 Max Delbrück Center for Molecular Medicine, Robert-Rössle-Str. 10, 13092 Berlin, Germany; 3 IRI Life Sciences, Institute für Biologie, Humboldt Universität zu Berlin, Philippstraße 13, 10115 Berlin, Germany; 4 Charité-Universitätsmedizin Berlin, 10117 Berlin, Germany; 5 Department of Biochemistry and Molecular Biology, University of Southern Denmark, Campusvej 55, 5230 Odense M, Denmark

## Abstract

Recruitment of the human ribonucleolytic RNA exosome to nuclear polyadenylated (pA^+^) RNA is facilitated by the Poly(A) Tail eXosome Targeting (PAXT) connection. Besides its core dimer, formed by the exosome co-factor MTR4 and the ZFC3H1 protein, the PAXT connection remains poorly defined. By characterizing nuclear pA^+^-RNA bound proteomes as well as MTR4-ZFC3H1 containing complexes in conditions favoring PAXT assembly, we here uncover three additional proteins required for PAXT function: ZC3H3, RBM26 and RBM27 along with the known PAXT-associated protein, PABPN1. The zinc-finger protein ZC3H3 interacts directly with MTR4-ZFC3H1 and loss of any of the newly identified PAXT components results in the accumulation of PAXT substrates. Collectively, our results establish new factors involved in the turnover of nuclear pA^+^ RNA and suggest that these are limiting for PAXT activity.

## INTRODUCTION

RNA turnover is a critical step in gene expression regulation and in the maintenance of cellular RNA homeostasis (1,2). The recent years utilization of high-throughput methods has noticeably broadened our known repertoire of RNA polymerase II (RNAPII)-derived transcripts. A large share of these is retained in the nucleus, where some play functional roles and others constitute labile by-products of pervasive transcription of the genome (3–5). Given such rich nuclear metabolism of long non-coding (lnc) RNA, it has become urgent to delineate pathways governing nuclear RNA decay. Here, the highly conserved nuclear RNA exosome stands out as a principal machinery involved in most nuclear RNA transactions (1,6,7).

The human nuclear exosome is composed of an inactive core that achieves its 3′–5′ exo- and endo-nucleolytic activities through interactions with the exonuclease RRP6 (EXOSC10) and the exo/endonuclease RRP44 (DIS3) (6,7). In addition, basal nuclear exosome function relies on RNA helicase activity, provided by the MTR4 (SKIV2L2) enzyme, which is essential for the unwinding of RNA substrates and for facilitating their threading through the exosome core to the nucleolytic activities (7). However, this is not enough. To obtain specificity and binding capacity toward its diverse set of substrates, the exosome utilizes MTR4 to engage with adaptor complexes (1,8). In the human nucleoplasm, two such adaptors have been described: (i) the Nuclear EXosome Targeting (NEXT) complex (9–11) and (ii) the PolyA Tail eXosome Targeting (PAXT) connection (12–14). For both of these mutually exclusive protein assemblies, MTR4 appears to mediate their critical connection to the RNA exosome. Within the NEXT complex, MTR4 associates with the zinc-knuckle protein ZCCHC8, which further couples to the RNA-binding protein RBM7 to facilitate the exosomal handover of short immature RNAs, such as PROMoter uPstream Transcripts/Upstream Antisense RNAs (PROMPTs/uaRNAs), enhancer RNAs (eRNAs) and 3′ extended products of snRNAs and snoRNAs (10,15–17). While MTR4, ZCCHC8 and RBM7 form a well-defined trimeric complex (11,18,19), what precisely constitutes the PAXT connection is presently elusive. It is clear that a tight and abundant dimer can form between MTR4 and the zinc-finger protein ZFC3H1, constituting the PAXT core (12,13). However, MTR4-ZFC3H1 can also form an RNA-dependent contact with the nuclear polyA binding protein (PABPN1), a connection that is critical for PAXT targeting of the exosome to longer and polyadenylated nuclear RNAs (12,20–22). Moreover, the ZFC3H1 interaction space is rather complex, comprising various proteins involved in nuclear RNA biology (12,23). It is therefore conceivable that additional factors may guide, or strengthen, PAXT interaction with its targets.

Our laboratory recently demonstrated that inactivation of the RNA exosome, by depletion of one of its core components, RRP40, causes the nuclear accumulation of polyA^+^ (pA^+^) RNA into distinct ‘pA^+^ RNA foci’ (14). Remarkably, PAXT components MTR4, ZFC3H1 and PABPN1 all co-localize with pA^+^ RNA foci, whereas NEXT components ZCCHC8 and RBM7 do not. We therefore suggested that PAXT substrates, and factor components, accumulate and coalesce into foci in the absence of RNA removal by the exosome (14). Moreover, ZFC3H1 is instrumental for pA^+^ RNA foci formation/maintenance as its co-depletion with RRP40 resulted in foci dissipation with some foci-retained transcripts being exported to the cytoplasm (13,14). Related aggregation of RNA with its decay targeting components has been reported to occur in the fission yeast *Schizosaccharomyces pombe* (*S. pombe*), where meiosis-specific transcripts form foci during mitotic growth of cells (24). These pA^+^ RNAs are targeted by the so-called MTl1-REd1 Core (MTREC) complex (25,26), also known as the NUclear RNA Silencing (NURS) complex (27). Interestingly, Mtl1 and Red1 are the *S. pombe* homologs of human MTR4 and ZFC3H1, respectively. Moreover, protein interaction studies revealed that MTREC associates with different sub-modules, one of which contains Pab2, the *S. pombe* homolog of human PAPBN1 (25). Altogether, this suggests functional similarities between MTREC and PAXT and reiterates the possibility that PAXT associates with additional yet-to-be-defined factors.

To identify additional PAXT-related factors in human cells, we here compare the nuclear pA^+^ RNA-bound proteomes from control and exosome compromised cells and reveal a clear enrichment of the zinc-finger protein ZC3H3 in the latter. Immunoprecipitation (IP) and transcriptome analyses further link ZC3H3 to PAXT biology. In alternative enrichment of the PAXT connection, by depleting the NEXT component ZCCHC8, we identify the putative RNA-binding proteins RBM26 and RBM27. Their co-depletion also impacts PAXT substrate levels and thus establishes RBM26 and RBM27, together with ZC3H3, as new components involved in the nuclear turnover of pA^+^ RNA. In conjunction, our data suggest that the RNA decay function of PAXT is mediated by a higher order protein assembly and not simply defined by the MTR4-ZFC3H1 dimer.

## MATERIALS AND METHODS

### Construct

For construction of the pcDNA5-ZC3H3–3xFLAG plasmid, the ZC3H3 CDS was PCR-amplified using forward primer TCA*GATATC*ATGGAGGAAAAGGAGATATTACG and reverse primer TCA*GCGGCCGC*CAGACGTGGTTTGATGTGCAG and cloned into the pcDNA5/FRT/TO-3xFLAG-C vector (28) using EcoRV and NotI restriction sites (italicized in primer sequence).

### Cell lines

HEK293 and HeLa cells were grown in DMEM containing 10% fetal bovine serum and 1% penicillin/streptomycin at 37°C and 5% CO_2_. The HeLa Kyoto cell line stably expressing LAP-tagged MTR4 was previously described (9). The HeLa Kyoto cell line stably expressing the LAP-only control was established as described in (29) using BAC clone CTD-3000G10.

### Cell line manipulation and construction

**Figure 1.**
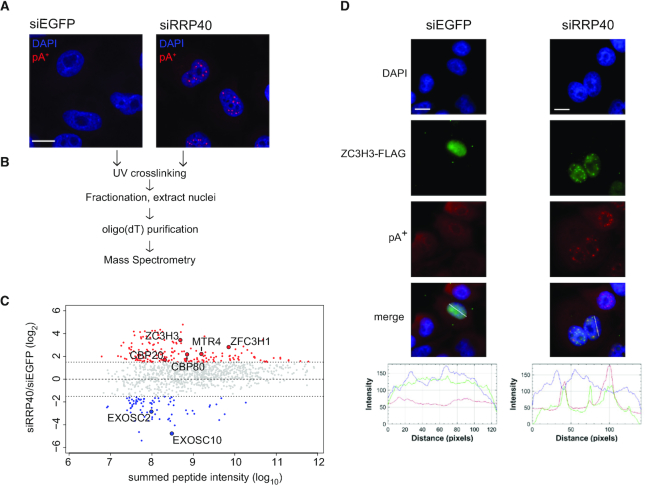
pA^+^-RNP purification from RRP40-depleted nuclei reveals enrichment of ZC3H3 in pA^+^ RNA foci. (**A**) RNA-FISH analysis of pA^+^ RNA in control (siEGFP) or RRP40-depleted (siRRP40) HeLa cells using a CY3-labeled oligo(dT)-LNA probe (47). pA^+^ RNA and DAPI signals are shown in red and blue, respectively; scale bar, 10 μm. (**B**) Schematic representation of the conducted pA^+^-RNP proteome purification. (**C**) MA plot representation of differentially purified pA^+^-RNP factors from RRP40-depleted versus control nuclei. Only proteins detected by 2 or more peptides in one of the samples are shown. Red and blue shadings indicate increased (log_2_(siRRP40/siEGFP) > 1.5) or decreased (log_2_(siRRP40/siEGFP) < −1.5) protein signals, respectively. *Y*-axis: log_2_(siRRP40/siEGFP), *X*-axis: log_10_ summed peptide intensities. Selected upregulated and downregulated proteins mentioned in the main text are highlighted. (**D**) Co-localization analysis of ZC3H3-FLAG and pA^+^ RNA in control (siEGFP) or RRP40-depleted (siRRP40) HeLa cells as indicated. ZC3H3-FLAG IF, pA^+^ RNA-FISH and DAPI stain are shown in green, red and blue, respectively. Line scan profiles represent IF, FISH and DAPI signal intensities along the drawn line shown on the merged picture panels; scale bars, 10 μm.

siRNA transfection was performed using Lipofectamine 2000 (Life Technologies) following the manufacturer’s instructions. Cells were seeded at low confluence (approximately 44 cells/mm^2^), and grown in DMEM for 24 h, before the first transfection. Cells were incubated in the presence of a final concentration of 20 nM of each siRNA (Supplementary Table S5) and Lipofectamine 2000 (final dilution, 1:1000) in RPMI 1640 medium. Two days after siRNA transfection, the medium was changed to DMEM without penicillin/streptomycin, and the transfection procedure was repeated. For co-localization experiments (e.g. Figure 1D), pcDNA5-ZC3H3–3xFLAG plasmid was transfected (using Lipofectamine 2000) into cells 1 day after the first siRNA transfection, and cells were harvested 1 day after the second siRNA transfection. HEK293 Flp-In T-Rex cells (Thermo Fisher Scientific) stably expressing ZC3H3–3xFLAG were established according to the manufacturer’s instructions. Expression of fusion protein was induced by replacing cell growth media with fresh media containing tetracycline (concentrations are indicated for each experiment separately).

### pA^+^ proteome capture

pA^+^-RNP capture was performed as in (30) with some modifications. Living Hela cells from control and RRP40 siRNA transfected cells (10^7^ cells per condition) were irradiated with 254 nm UV light (0.15 J/cm^2^) and collected to 50 ml tubes by scraping in cold PBS. Collected cells were fractionated according to the protocol in (14). Extracted nuclei were lysed in 10 ml of ice-cold lysis buffer (20 mM Tris-HCl pH 7.5, 500 mM LiCl, 0.5% LiDS, 1 mM EDTA and 5 mM DTT, 40 U RiboLock RNase inhibitors (Thermo Fisher Scientific) and 1× protease inhibitors (Roche)) by pipetting. For homogenization, lysates were passed through a syringe with a narrow needle (gauge 1.2 mm diameter 3–5 times, then gauge 0.8 mm diameter 3–5 times, finally gauge 0.5 mm diameter 3–5 times). Prepared lysates were incubated for 10 min on ice. Protein concentration was measured by Bradford assay (Bio-Rad) and equal amounts of total nuclear protein were used for subsequent pull-downs. Before pA^+^ RNA pull-down, lysates were spiked with 10% of Hela nuclear extracts from cells grown in SILAC heavy isotope medium. About 1 ml of oligo(dT)_25_ magnetic beads (Thermo Fisher Scientific, Dynabeads, Cat. no. 61005) were equilibrated in three volumes of lysis buffer and added to the nuclear lysate and incubated for 1 h at 4°C with gentle rotation. Tubes were placed on a magnet at 4°C and until the beads were completely captured on the tube wall. Supernatants were transferred into a new tube and kept on ice until used in two additional cycles of oligo(dT) capture. Combined captured beads were then washed two times in 10 ml of ice-cold lysis buffer and two times in 10 ml of ice-cold NP40 wash buffer (50 mM Tris-HCl pH 7.5, 140 mM LiCl, 2 mM EDTA pH 8.0, 0.5% NP40, 0.5 mM DTT) by incubating for 5 min at 4°C with gentle rotation. Beads were collected using a magnet and supernatants, after each wash step, were discarded. Captured RNA–protein complexes were eluted with 50 μl of elution buffer (10 mM Tris-HCl pH 7.5) for 2 min at 80°C. After elution, beads were transferred to lysates saved from previous rounds of capture. Finally, eluates from three successive cycles of pA^+^ RNA capture were pooled (final volume 150 μl). About 23 μl of eluate was RNase treated with an A1/T1 mix and used for western blotting analysis and silver staining. The remaining eluates were used for MS analysis as described in (30). Potential contaminants were removed and proteins detected in the SILAC light fraction (i.e. pA^+^ RNA capture samples) in both siEGFP and siRRP40 conditions were selected for further analysis (*n* = 1393). Protein intensities from the SILAC heavy fraction (i.e. spike-ins) in the siEGFP and siRRP40 samples were used to compute a scaling factor with the estimateSizeFactorsForMatrix function from R package DESeq2 (v1.20) using default settings. Protein intensities from the light fraction were then divided by obtained size factors and log_2_ ratio between normalized intensities from siRRP40/siEGFP samples calculated (Supplementary Table S1). Values from proteins detected with two or more peptides in either siRRP40 or siEGFP samples were plotted against the sum of normalized intensities from both conditions (Figure 1C).

### GO terms

Proteins with a log_2_(siRRP40/siEGFP) > 1.5 were considered specifically enriched in siRRP40 samples. GO terms associated with those proteins were determined using R package clusterProfiler (v3.10.1) and human proteome annotations and GO terms from R package org.Hs.eg.db (v3.7.0). Terms associated with enriched proteins were determined using clusterProfiler function enrichGO with settings pAdjustMethod = ‘BH’, pvalueCutoff = 0.01, qvalueCutoff = 0.05 using the human proteome as background. Analysis was done for ontology categories cellular component (CC) and biological process (BP).

### Western blotting analysis

Cell pellets were re-suspended in RSB100 (100 mM Tris-HCl pH 7.5, 100 mM NaCl, 2.5 mM MgCl_2_, 0.5% Triton X-100) followed by incubation on ice for 10 min. Cell debris was removed by centrifugation at 4000*g*, 4°C for 15 min. Samples were separated by 4–12% NuPAGE Novex Bis-Tris or 3–8% Tris-Acetate gels and transferred to PVDF membranes (Millipore), which were blocked in 5% skimmed-milk powder (SMP) in PBS for 1 h at room temperature. Primary antibody was added to 5% SMP in PBS and membranes were incubated 1 h at room temperature, followed by three washes for 5 min in PBS/0.05% Tween 20 and incubated in horseradish peroxidase (HRP)-conjugated goat-anti-rabbit or -mouse secondary antibody (Dako) in 5% SMP in PBS. Thereafter, membranes were washed three times for 5 min in PBS/0.05% Tween 20 and exposed using Supersignal West Femto Substrate (Thermo Fischer Scientific). All used antibodies and the applied concentrations are listed in Supplementary Table S6 (31).

### α-FLAG IP

Expression of ZC3H3-FLAG was induced in HEK293 cells by the addition of 2.5 ng/ml tetracycline. Twenty-four hours after induction, media were removed and cells (1.5 × 10^7^) were washed once with cold PBS and collected in a tube by scraping the cells in 1 ml of cold PBS, containing 3 mM EDTA. Cells were then pelleted by centrifugation and immediately resuspended in lysis buffer (150 mM NaCl, 50 mM Tris-HCl pH 7.5, 5% glycerol, 1% IGEPAL-CA-630, 1 mM MgCl_2_) supplemented with 1× protease inhibitors (Roche). Cell suspension was incubated for 10 min on ice and sonicated twice for 10 s at 20 W using a Branson 250 sonicator. Lysates were cleared by centrifugation at 4°C and protein extracts transferred to a new tube. Protein concentrations were measured by Bradford assay and samples adjusted to equal protein contents. 10% of adjusted lysates were saved and used for input analysis. Remaining lysates were used in IP reactions with α-FLAG magnetic beads. Beads were prepared by coupling 500 μg of α-FLAG antibody (clone M2, mouse, Sigma #F3165) to 75 mg of Dynabeads M-270 Epoxy (Life Technologies) for 1 h under gentle rotation in the cold room. After binding, beads were washed three times with lysis buffer, two times with wash buffer I (150 mM NaCl, 50 mM Tris-HCl pH 7.5, 5% glycerol and 0.05% IGEPAL-CA-630) and one time with extraction buffer (100 mM NaCl, 20 mM HEPES-KOH pH 7.4, 0.5% Triton). After the final wash, beads were resuspended in extraction buffer and divided into three tubes for BSA (1 mg/ml) and nuclease treatments with the 5 U RNase T1 and 2 μg RNase A mix (Thermo Scientific #EN0551) and 250 U Benzonase (Sigma E1014–5KU) for 20 min at room temperature with gentle agitation (1000 rpm, Eppendorf thermomixer). After incubation, beads were collected on a magnetic rack and supernatants (RNase released proteins) were transferred to new tubes. Beads were then washed twice with wash buffer I and bead-bound proteins were eluted with 1.5× LDS in water by incubating 10 min at 75°C with continuous mixing. Eluates and inputs were analyzed by SDS-PAGE followed by western blotting analysis. About 3% of the initial input material and 30% of the FLAG eluate, respectively, were loaded per lane.

### RNA isolation and RT-qPCR analysis

RNA was purified from freshly cultured cells using TRIzol reagent (Thermo Fisher Scientific) according to the manufacturer’s instructions. Quantification and quality assessment of RNA were done by spectrophotometry (NanoDrop; Thermo Scientific) and gel electrophoresis. Purified RNA was DNase I treated (TURBO DNA-free Kit, Thermo Fisher Scientific) and for RT-qPCR analysis cDNA was synthesized using 1 μg of DNase-treated RNA and random hexamer primers with SuperScript III Reverse Transcriptase according to the manufacturer’s instructions (Thermo Fisher Scientific). qPCR was performed in a 20 μl reaction volume using Platinum® SYBR® Green qPCR SuperMix-UDG (Thermo Fisher Scientific) according to the manufacturer’s instructions with primers listed in Supplementary Table S7 on an AriaMx Real-time qPCR System (Agilent). Data were processed using the ΔΔCt method, with normalization to both GAPDH mRNA levels and EGFP-siRNA control samples.

### RNA-FISH/IF analysis

pA^+^ RNA FISH was performed as recently described (14). For co-staining of RNA with protein, RNA-FISH reactions were followed by protein immunolocalization analysis. Briefly, a primary antibody dilution in 2% BSA was added to cells for 1 h at room temperature, followed by three brief washes with PBS. Thereafter, Alexa-Fluor 488-conjugated secondary antibody (Thermo Fisher Scientific) in 2% BSA was added for 1 h at room temperature, followed by three brief washes with PBS. Cells were mounted with ProLong Gold Antifade Mountant with DAPI. Primary antibody dilutions were the following: anti-FLAG (Sigma-Aldrich, #F3165) 1:2000, anti-ZFC3H1 (Novus Biologicals, NB100–68267) 1:500, anti-PABPN1 (Abcam, ab75855) 1:1000.

### LAP-tag IP-MS

IP-MS experiments were performed label-free and in triplicates, essentially as described (9,12,28). Briefly, MTR4-LAP and control-LAP cells were resuspended in extraction buffer (20 mM HEPES-KOH pH 7.4, 0.5% TritonX-100, 150 mM NaCl) supplemented with 1× protease inhibitor (Roche), sonicated three times for 3 s with a Branson 250 sonicator, followed by centrifugation at 14 000*g*, 4°C for 10 min. The supernatant was incubated with anti-GFP llama polyclonal antibodies (conjugated to Dynabeads Epoxy M270 (Invitrogen) as previously described (28)), rotating at 4°C for 30 min. Beads were then washed three times with extraction buffer and two times with 1 ml 20 mM Tris-HCl pH 8.0, 2 mM CaCl_2_ before ‘on beads’ trypsin digestion. Beads were then resuspended in 50 μl 20 mM Tris-HCl pH 8.0 with 750 ng trypsin (Promega V5111) and incubated overnight at 37°C. The supernatant was further digested with additional 250 ng trypsin for 3 h at 37°C. The samples were then acidified with formic acid (final concentration of 2%) and centrifuged at 16 000*g* for 5 min to remove debris. The supernatant was snap frozen and stored at −80°C before MS analysis. Prior to MS analysis, sample was acidified with 1% trifluoroacetic acid (TFA) to pH ∼ 1, and loaded onto C18 stage tips that were washed with methanol, pre-equilibrated with 1% TFA and then washed with 1% TFA. Peptides were eluted with 80% acetonitrile (ACN), 0.5% acetic acid and dried in a speedvac. Samples were resuspended in 2% ACN, 0.1% TFA, then analyzed by a Q exactive HF mass spectrometer. MS data were processed with Maxquant and the Perseus package. Log_2_ transformed LFQ intensities were used for further analysis with the statistical software package R. A Student’s *t* test (R’s t.test default settings: two-sided, unpaired, assuming non-equal variance) was used to obtain the absolute difference and *P*-value for significance of the difference between triplicate MTR4-LAP versus triplicate control IPs. This was done separately for siLUC conditions and siZCCHC8 conditions depicted as volcano plots in Supplementary Figures S3A and S3B, respectively. For comparison between siLUC and siZCCHC8 conditions, proteins detected significantly (*P* < 0.1, log_2_(MTR4-LAP/Control) > 0) in either siZCCHC8 or siLUC were selected and then all log_2_LFQ values were first normalized by subtracting the log_2_LFQ for MTR4 in the same sample (i.e. norm.log_2_LFQ_MTR4_ = 0). The normalized data were then used for Student’s *t* test between siZCCHC8 and siLUC for the MTR4-LAP conditions.

### Glycerol gradient sedimentation analysis

Glycerol gradient sedimentation analysis was performed as described in (32) with some modifications. Expression of ZC3H3-FLAG was induced in HEK293 cells by the addition of 2.5 ng/ml tetracycline. Twenty-four hours after induction, media were removed and cells were washed once with cold PBS and harvested by trypsinisation. Cells (∼2 × 10^8^) were resuspended in BC100 buffer (33) (5 mM HEPES pH 7.5, 100 mM NaCl, 1 mM MgCl_2_, 0.5 mM EGTA, 0.1 mM EDTA, 10% v/v glycerol, 1 mM DTT) supplemented with protease inhibitors, lysed by sonication (3 × 5 s, amplitude 2) and centrifuged at 14000 rpm for 20 min. Clarified lysates were loaded on 5–30% glycerol gradients prepared in BC100 buffer and centrifuged at 35000 rpm for 24 h using a SW41 rotor (Beckman). Gradients were separated into 18 fractions and protein content was assessed using the Bradford assay (Bio-Rad). The remaining fractions were TCA precipitated, acetone washed and resuspended in 1× NuPAGE loading buffer (Thermo Fisher Scientific). Samples were separated by SDS-PAGE and analyzed by western blotting.

### RNAseq: source data

Raw reads from total RNA of siEGFP, siZCCHC8, siZFC3H1 and siRRP40 RNAseq libraries from (12) (SRA:SRP078134) were processed in parallel with siZC3H3 and siRBM26/siRBM27 libraries reported the first time here deposited at GEO: GSE131255. All data from (12) were considered one batch (‘batchMeola’). The other libraries were generated from total RNA using BGIs long non-coding RNA sequencing service and sequenced using paired-end sequencing of 100 nt reads. siZC3H3 libraries were prepared together with triplicate siEGFP controls and was considered one batch (‘batchZC3H3’). siRBM26/RBM27 libraries were based on RNA samples prepared in two different experiments with replicate 1 and 2 stemming from 2+1 day depletions, whereas replicate 3 was from a 2+2 day depletion, with each replicate being processed in parallel with siEGFP controls. Replicates 1 and 2 behaved very similarly and were treated as 1 batch (‘batchRBM’), whereas replicate 3 showed clear batch-specific differences (data not shown) and was thus treated as a separate batch (‘batchRBM.2’). Note that despite clear batch-specific differences between libraries, relevant biological effects were visible in all individual depletion experiments irrespective of batch correction (data not shown).

### RNAseq: filtering and mapping

Raw reads were quality filtered and trimmed as described (12), using Trimmomatic (v0.32) and settings (PE ILLUMINACLIP:/com/extra/Trimmomatic/0.32/adapters/TruSeq3-PE-2.fa:2:30:10 HEADCROP:12 LEADING:22 SLIDINGWINDOW:4:22 MINLEN:25). Cleaned reads were then mapped to hg38 with HISAT2 (v2.1.0) using default settings and the genome index ‘H. sapiens, UCSC hg38 and Refseq gene annotations’ provided at the HISAT2 download page (ftp://ftp.ccb.jhu.edu/pub/infphilo/hisat2/data/hg38_tran.tar.gz). Only proper pairs with both reads mapping to the genome were used for further analysis. Strand-specific coverage tracks were then computed using the genomeCoverage function from bedtools and coverage normalized to the total number of uniquely mapped and properly paired reads and converted to bigwig format using UCSC tools. Individual normalized bigwig files are available at GEO: GSE131255. Genome Browser Tracks show the average normalized signals from three biological replicates at each position.

### RNAseq: transcript-level counting, clustering and differential expression

Exon-specific read counts were obtained from uniquely mapped and properly paired reads from bam files using htseq-count (HTSeq framework v0.6.0) and settings [-f bam -s reverse -t exon -m intersection-strict], and Refseq annotations for hg38 (RefSeqNCBIAll_GRCh38.gtf, obtained from UCSC Genome Browser using the Table Browser function). Obtained counts were then analyzed by the statistical software R with package DESeq2 (v1.20.0), using default settings and a design formula, that included batch information as described above (design = ∼ siRNA + batch). For clustering of read count matrices, raw DESeq2 objects were subjected to variance stabilizing transformation (using vst function from DESeq2) and batch effects in the transformed count matrix were balanced using the removeBatchEffect functions from R package limma (v3.36.1), providing batch information as specified above. Processed read counts were then used to compute pearson correlations and euclidian distances between samples. Heat maps of Pearson correlations and clustering of distances between samples were depicted using the R function hclust using distance = ward.D2 (Supplementary Figure S4A). For PCA analysis (Supplementary Figure S4B), the processed read count matrix was subject to the plotPCA function from DESeq2. Differentially expressed genes within each depletion sample were computed using DESeq2. Transcripts significantly (padj < .1) upregulated in siZFC3H1 or siZCCHC8 conditions were grouped into genes with higher upregulation in siZFC3H1 (‘PAXT’) or higher upregulation in siZCCHC8 (‘NEXT’).

### RNAseq: PROMPT profiles

Transcription Start Sites (TSSs) of PROMPTs from (34) were lifted to hg38 and used for metagene analysis of 100 bp bins for regions −1000 to +4000 bp relative to the TSS, using a custom scripts based on deeptools (v3.0.2, (35)). For further analysis, a pseudocount (the smallest positive value among all bins) was added to all bins and the log_2_(KD/siEGFP) computed for bins. PROMPTs were grouped using the mean log_2_ fold change (computed as above) between siZCCHC8 and siZFC3H1 samples in the region from TSSs to +500 bp. PROMPTs with mean log_2_FC (siZFC3H1/siEGFP) > 0 and mean log_2_FC (siZFC3H1/siZCCHC8) > 0.5 were classified as PAXT dependent and PROMPTs with mean log_2_FC (siZCCHC8/siEGFP) > 0 and mean log_2_FC (siZFC3H1/siZCCHC8) < −0.5 were classified as NEXT dependent. Figure 4C shows mean log_2_FC and 95% confidence intervals for the mean computed for both classes and all positions.

### Statistical analysis

Values of biological replicates and statistical tests are reported in each of the relevant figure legends. qRT-PCR data are shown as means ± SDs. Statistical tests were conducted in the environment of the R Project for Statistical Computing (https://www.r-project.org).

## RESULTS

### Nuclear pA^+^ RNP characterization of exosome depleted cells identifies ZC3H3

We recently demonstrated that depletion of the core RNA exosome component RRP40 triggers the accumulation of pA^+^ RNA in distinct nuclear foci, which we recapitulated (Figure 1A and Supplementary Figure S1A, (14)). As PAXT components MTR4, PABPN1 and ZFC3H1 localize to the same foci (14), we decided to search for additional factors involved in pA^+^ RNA metabolism by purifying the nuclear pA^+^ RNP proteome. To this end, we UV-irradiated control or RRP40-depleted HeLa cells to induce RNA-protein cross-links (30,36) and subsequently used extracted nuclei (Figure 1B and Supplementary Figure S1B) to capture pA^+^ RNP proteomes onto oligo(dT) beads. After stringent washes, remaining proteins were identified by mass-spectrometry (MS) (Figure 1C and Supplementary Figure S1C). Comparison of pA^+^ RNP proteomes from control versus RRP40-depleted nuclei revealed the enrichment of 208 proteins (Figure 1C; Supplementary Figure S1C and Supplementary Table S1). Gratifyingly, MTR4 and ZFC3H1 were enriched upon RRP40 depletion, whereas detected exosome proteins were depleted, which validated the approach. Moreover, gene ontology (GO) analysis showed the overrepresentation of factors related to nuclear RNA processing and export, consistent with enrichment of proteins bound to pA^+^ RNA (Supplementary Table S2).

Another protein, ZC3H3, which was enriched in the pA^+^ RNP proteome from RRP40-depleted nuclei, caught our attention, as it is the human homolog of the *S. pombe* Red5 protein, previously shown to be part of an MTREC sub-module (25,27). To confirm that ZC3H3 localizes to pA^+^ RNA foci, we expressed FLAG-tagged ZC3H3 exogenously in control and RRP40 depleted cells. Simultaneous pA^+^ RNA-fluorescence *in situ* hybridization (FISH) and FLAG-immunofluorescence (IF) analyses revealed a marked co-localization of signals in RRP40 deplete conditions (Figure 1D). Taken together, this demonstrated that nuclear pA^+^ RNPs can be purified for proteome identification by MS and that this tactic identified ZC3H3 as an interesting candidate for further study.

### ZC3H3 interacts physically and functionally with PAXT components

**Figure 2.**
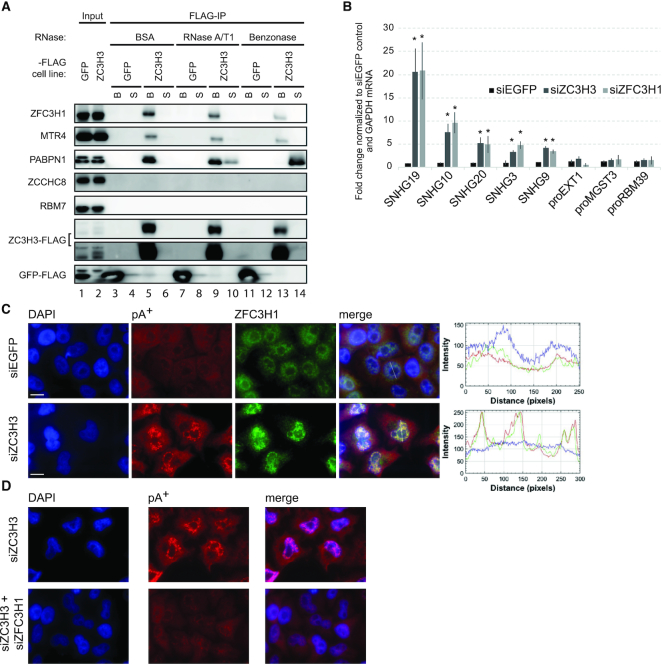
ZC3H3 is physically and functionally linked to the PAXT connection. (**A**) Western blotting analysis inquiry of whether the indicated NEXT and PAXT related proteins co-purify with exogenously expressed ZC3H3-FLAG fusion protein. Anti-FLAG beads were used to capture ZC3H3-FLAG complexes, which were divided in three batches and treated with BSA (control), RNase A/T1 or Benzonase, respectively. Nuclease (or BSA) released supernatants (S) and bead-bound (B) counterparts are shown. Moreover, 3% of input samples are shown in lanes 1 and 2. A GFP-FLAG IP was conducted as a negative control experiment. Note that PABPN1 was also not detectable in Benzonase-treated samples upon longer exposure of the represented western blot (data not shown). (**B**) RT-qPCR analysis of PAXT substrates SNHG19, −10, −20, −3, −9 and NEXT substrates proEXT1, proMGST3 and proRBM39 using total RNA harvested from control (siEGFP), ZC3H3-depleted (siZC3H3) and ZFC3H1-depleted (siZFC3H1) HeLa cells. Data are displayed as mean values, with error bars denoting SD (*n* = 3 biological replicates). ∗ *P* < 0.05, Student’s *t* test. (**C**) Co-localization analysis of ZFC3H1 and pA^+^ RNA in control (siEGFP) or ZC3H3-depleted (siZC3H3) HeLa cells. Image organization as in Figure 1D. (**D**) FISH analysis of pA^+^ RNA in ZC3H3-depleted (siZC3H3) and ZC3H3/ZFC3H1 double-depleted (siZC3H3+siZFC3H1) HeLa cells as in Figure 1A.

Given the physical connection between Red1 (ZFC3H1) and Red5 (ZC3H3) in *S. pombe*, we next interrogated ZC3H3 interactions, focusing on NEXT and PAXT components. To this end, we generated HEK293 cells stably expressing tetracycline-inducible ZC3H3-FLAG protein (Supplementary Figure S2A) and IP’ed interacting proteins using an anti-FLAG antibody. The bead-bound material was treated with BSA (control), RNase A/T1 (which does not degrade DNA and stretches of A-residues (37)) or Benzonase (which degrades all forms of DNA and RNA (38)) before final elution. Western blotting analysis of the respective eluates revealed that ZC3H3 precipitated PAXT components MTR4, ZFC3H1 and PABPN1, whereas the GFP-FLAG control did not (Figure 2A, lanes 3 and 5). In contrast, NEXT components RBM7 and ZCCHC8 were not present in FLAG-ZC3H3 IPs. Importantly, ZC3H3’s interaction with PABPN1 was partly and completely abolished in the presence of RNase A/T1 and Benzonase, respectively (Figure 2A, compare lanes 9 to 10 and 13 to 14), whereas its interactions with MTR4 and ZFC3H1 were only mildly affected upon nuclease treatments. This echoes the ZFC3H1–PABPN1 and ZFC3H1–MTR4 interactions, which are similarly RNA-dependent and independent (12). However, we note that a lower fraction of the input MTR4 and ZFC3H1 was recovered in the FLAG-ZC3H3 IP, compared to the more efficiently precipitated PABPN1 (Figure 2A, compare ‘Input’ and ‘FLAG-IP’ lanes). This suggests that connecting ZC3H3–PABPN1 and MTR4–ZFC3H1 modules also form separate interactions, which are RNA-dependent and -independent, respectively. Moreover, it implies that a significant portion of the nuclear ZFC3H1–MTR4 dimer might not be engaged with ZC3H3 and PABPN1 (see ‘Discussion’ section).

Having established a physical link between ZC3H3 and PAXT components, we next investigated whether ZC3H3 depletion would affect the abundance of known PAXT targets (12). To this end, HeLa cells were transfected with ZC3H3 specific siRNAs, resulting in ZC3H3 mRNA and protein depletion, respectively (Supplementary Figure S2B and S2C). As the used ZC3H3 antibody generated unspecific bands in HeLa cells, we further tested the ZC3H3 siRNA and antibody specificity in our tetracycline-inducible ZC3H3-FLAG HEK293 cells. This confirmed that the ZC3H3 antibody recognizes the ZC3H3-FLAG protein and that the ZC3H3 siRNA is functional (Supplementary Figure S2A). Further analysis of known PAXT targets, the spliced products of snoRNA host genes (SNHGs), showed that their levels increased in ZC3H3 depleted cells comparably to those in ZFC3H1 depleted cells, whereas levels of NEXT complex PROMPT targets, proEXT1, proMGST3 and proRBM39, did not significantly change upon neither ZC3H3 nor ZFC3H1 depletion (Figure 2B). Finally, we tested whether ZC3H3 depletion would lead to pA^+^ RNA foci formation as previously observed upon exosome inactivation (14). This was indeed the case, although the foci appeared smaller and more plentiful (compare Figure 2C with 1D). However, as also described for RRP40 depleted cells, the formed foci were enriched for ZFC3H1 protein and PABPN1 (Figure 2C and Supplementary Figure S2D), and they were substantially reduced upon co-depletion of ZFC3H1 (Figure 2D). We also used specific RNA-FISH analysis to localize the PAXT target SNHG19 upon ZC3H3 depletion, which revealed its localization within pA^+^ RNA foci as previously observed upon exosome depletion (Supplementary Figure S2E, (14)). Altogether these results underscore a central role for ZC3H3 in nuclear exosome-mediated RNA turnover and at the same time uncover a functional specialization within PAXT, where ZC3H3 is required for RNA decay of PAXT targets but not their nuclear aggregation, contrasted by ZFC3H1, which is essential for both decay and aggregation.

### The RNA-binding proteins RBM26/RBM27 are functionally connected to PAXT

**Figure 3.**
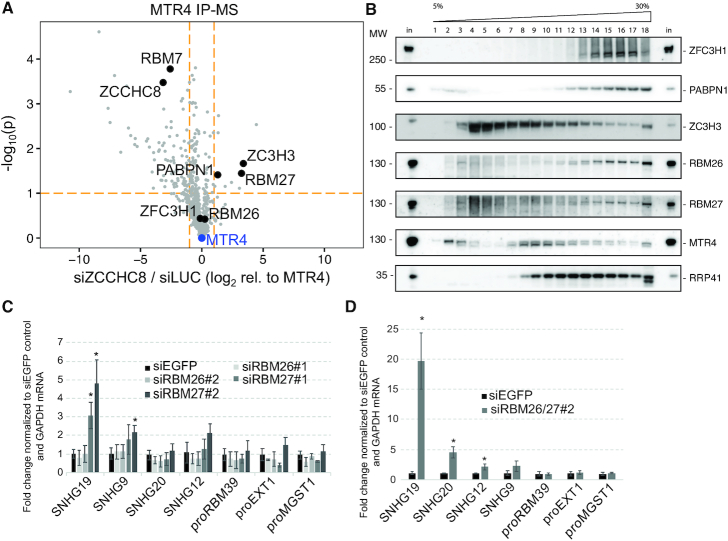
RBM26 and RBM27 harbor PAXT activity. (**A**) Comparison of protein abundance (label-free quantification (LFQ) values) in triplicate hMTR4-LAP affinity capture mass spectrometry (AC-MS) experiments from ZCCHC8-depleted (siZCCHC8) to control (siLUC) HeLa Kyoto cells. Volcano plot depicts the ratio of protein abundances (log_2_(LFQ)) normalized to the MTR4 bait from siZCCHC8 versus siLUC AC-MS experiments (*X*-axis) and the -log_10_ of the *P*-values from a Student’s *t* test for this comparison. Only proteins significantly enriched relative to a control AC-MS in either siLUC or siZCCHC8 (see Supplementary Figure S3A and S3B) are depicted here. Relevant proteins are highlighted and labeled. Orange dashed lines mark log_2_FC = -/+ 1 and -log_10_*P*-values = 1. (**B**) Glycerol gradient (5–30%) western blotting analysis of the sedimentation of the indicated proteins from extracts of HEK293 ZC3H3-FLAG protein expressing cells. Fractions of input (‘in’) were loaded on first and last lanes for comparison. (**C**) RT-qPCR analyses of PAXT and NEXT substrates as in Figure 2B but using total RNA harvested from RBM26-depleted (siRBM26), RBM27-depleted (siRBM27) or control (siEGFP) HeLa cells. Results of two independent RBM26 and RBM27 siRNAs (indicated as #1 and #2) are shown. Data are displayed as mean values, with error bars denoting SD (*n* = 4 biological replicates). ∗ *P* < 0.05, Student’s *t* test. (**D**) RT-qPCR analyses of PAXT and NEXT substrates as in panel (C) using total RNA harvested from RBM26/RBM27 (siRBM26/27) co-depleted or control (siEGFP) HeLa cells. Note that for co-depletion only siRNAs #2 were used. Data are displayed as mean values, with error bars denoting SD (*n* = 3 biological replicates). ∗ *P* < 0.05, Student’s t test.

MTR4 forms mutually exclusive interactions with ZCCHC8 (NEXT) and ZFC3H1 (PAXT) (12). We therefore surmised that lowering nuclear ZCCHC8 levels might lead to the enrichment of proteins relevant for the PAXT-MTR4 interactome. Hence, we depleted ZCCHC8 in HeLa cells stably expressing a ‘localization and affinity purification’ (LAP) tagged MTR4-LAP fusion protein (9,12) and performed IP with GFP antibody recognizing the LAP-tag followed by the identification of any captured proteins by MS (Supplementary Table S3). As expected, known NEXT and PAXT components were readily revealed in MTR4 IPs conducted from cells treated with a control siRNA (Supplementary Figure S3A). Moreover, treatment with a ZCCHC8-specific siRNA resulted in reduced MTR4 interaction of both ZCCHC8 and RBM7, whereas levels of PAXT components PABPN1 and ZC3H3 were increased (Figure 3A and Supplementary Figure S3B). Interestingly, ZCCHC8 depletion also led to an increased interaction of MTR4 with the RNA-binding protein RBM27 (Figure 3A), which conspicuously is the human homolog of the *S. pombe* Rmn1 protein, suggested to reside in an MTREC sub-module with Red5 (ZC3H3 ortholog) and Pab2 (PABPN1 ortholog) (25). A similar sub-module may therefore exist in human cells and we conclude that the MTR4-ZFC3H1 dimer is capable of interacting with RBM27, PABPN1 and ZC3H3. The other eight proteins enriched upon ZCCHC8 depletion (Supplementary Table S3) have no obvious connection to PAXT biology and were not analyzed further. Moreover, we note that ZCCHC8 depletion had largely no impact on the amount of ZFC3H1 co-precipitated with MTR4 (Figure 3A). This corroborates our previous note (Figure 2A) that a major fraction of nuclear ZFC3H1 presumably resides in a dimer with MTR4, and which is formed equally efficient in the presence or absence of the NEXT complex. In contrast, MTR4’s interaction with the uncovered higher order PAXT connection, comprising additionally ZC3H3, RBM27 and PABPN1, appears to occur in competition with NEXT (see ‘Discussion’ section).

To further elaborate on the potential connection between ZC3H3, RBM27 and its paralog RBM26 (39) with PAXT and the exosome core, we performed glycerol sedimentation analysis of extracts from FLAG-ZC3H3 expressing HEK293 cells. The RNA exosome core component RRP41, PAXT proteins ZFC3H1, MTR4 and PABPN1 as well as ZC3H3, RBM27 and RBM26 mostly sedimented in the high molecular weight fractions (Figure 3B, lanes 12–18). However, we also note that MTR4 and ZC3H3 were present in low molecular weight fractions (Figure 3B), which for ZC3H3 was likely due to an excess of free ZC3H3 protein due to its exogenous overexpression Supplementary Figure S2A). Altogether, these results are consistent with the possibility that RBM27, RBM26 and ZC3H3 are assembling with PAXT and the RNA exosome.

Having linked RBM27 and RBM26, together with ZC3H3 and PABPN1, to a fraction of the MTR4-ZFC3H1 dimer, we next inquired whether RBM27 and RBM26 might affect PAXT functionality. To this end, we designed two sets of siRNAs targeting RBM27 or RBM26, and confirmed their efficiencies in depleting the respective mRNAs (Supplementary Figure S3C). Western blotting analysis using an available RBM27 antibody corroborated the relevant RT-qPCR result (Supplementary Figure S3D). Subsequent transcript analyses revealed that levels of NEXT-specific PROMPTs remained largely unchanged upon both RBM26 and RBM27 depletion (Figure 3C). In contrast, 3 of 4 tested spliced products of SNHGs were upregulated in RBM27 depleted cells. To interrogate whether RBM26 and RBM27 may have partially redundant functions in PAXT substrate targeting, we conducted a co-depletion experiment, which revealed that doubly RBM26 and RBM27-depleted HeLa cells exhibited a synergistic upregulation of 2 of the 4 tested SNHG transcripts (SNHG19 and SNHG20), whereas NEXT substrates were still unaffected (Figure 3D). We conclude that RBM26 and RBM27 act redundantly and will refer to these proteins as RBM26/RBM27 below. Moreover, it appears that two sets of RNA-binding proteins, RBM26/RBM27 and PABPN1, are involved in PAXT-dependent RNA decay.

### ZC3H3, RBM26 and RBM27 links to PAXT biology are general

**Figure 4.**
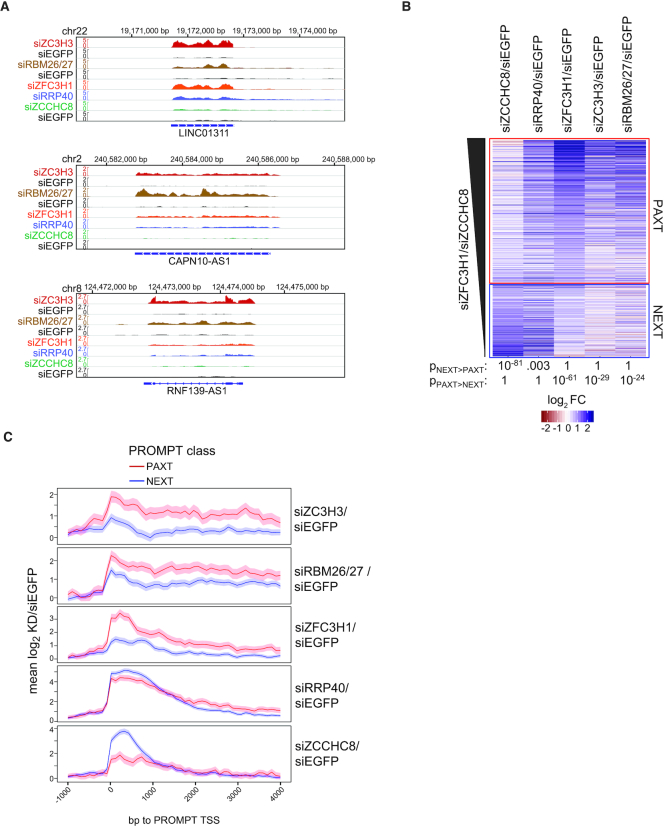
Genome-wide analyses of ZC3H3, RBM26 and RBM27 targets. (**A**) Genome browser screenshots of lncRNA loci LINC01311, CAPN10-AS1 and RNF139-AS1, displaying RNAseq signals from siZC3H3, siRBM26/27, siZFC3H1, siRRP40 and siZCCHC8 samples as well as their respective siEGFP controls. siZC3H3 and siRBM26/27 samples were produced in separate batches and are shown on top of their same-batch siEGFP controls. Only the strands expressing the respective RNAs are shown. All data were normalized to genome-wide coverage and are shown on the same scale as the respective control as indicated. (**B**) Heat map representation of log_2_ fold changes of individual transcripts within the indicated depletion libraries relative to their siEGFP controls. Shown are transcripts, which were significantly upregulated in siZCCHC8 or siZFC3H1 libraries and sorted by their siZFC3H1/siZCCHC8 ratios and grouped into primarily ‘PAXT’ (siZFC3H1 > siZCCHC8, *n* = 718) or ‘NEXT’ (siZFC3H1 < siZCCHC8, *n* = 362) sensitive. Wilcoxon rank sum test *P*-values for significance of differences in log_2_ fold changes between NEXT and PAXT classes are listed below the heat map. (**C**) Metagene profiles for PROMPTs classified as ‘NEXT’ (primarily siZCCHC8 sensitive, *n* = 574) or ‘PAXT’ (primarily siZFC3H1 sensitive, *n* = 262). Shown are mean log_2_ fold changes with 95% confidence intervals of the indicated depletion libraries relative to their siEGFP controls for regions −1 kb to + 4 kb around PROMPT TSSs (34).

Having demonstrated that ZC3H3 and RBM26/RBM27 proteins impact levels of selected PAXT targets, we investigated the generality of this observation by preparing triplicate RNAseq libraries of rRNA-depleted samples from cells depleted for ZC3H3 (siZC3H3), or co-depleted for RBM26/RBM27 (siRBM26/27), and compared these to data from our previously published NEXT (siZCCHC8), PAXT (siZFC3H1) and RNA exosome (siRRP40) depletion RNAseq data sets (12). After removal of batch effects (see ‘Materials and Methods’ section), each depletion library revealed an apparent specific expression signature (Supplementary Figure S4A and S4B). The set of significantly upregulated transcripts in both siZC3H3 and siRBM26/27 libraries largely contained lncRNA biotypes, such as antisense RNAs and long intergenic non-coding (linc) RNAs (Supplementary Table S4), indicating nuclear exosome co-factor functions of ZC3H3 and RBM26/27. Consistently, genome browser views displayed clear increases of selected lincRNAs in siZFC3H1, siZC3H3 and siRBM26/siRBM27 libraries (Figure 4A).

To create a global comparison of the different depletion libraries, we next selected transcripts significantly upregulated in siZCCHC8 or siZFC3H1 libraries, respectively, and classified those more sensitive to siZFC3H1 than siZCCHC8 as ‘PAXT’ dependent and those more sensitive to siZCCHC8 than siZFC3H1 as ‘NEXT’ dependent. Despite some overlap between siZFC3H1 and siZCCHC8 targets, this division demonstrated that PAXT sensitive transcripts were generally more sensitive to ZC3H3 and RBM26/RBM27 depletions than NEXT sensitive transcripts (Figure 4B). This was in line with an unsupervised clustering of the data, separating siZCCHC8 data from the siZC3H3, siRBM26/27 and siZFC3H1 data (Supplementary Figure S4A). Thus, at the genome-wide scale siZC3H3 and siRBM26/27 affected transcripts overlap those impacted by depletion of the PAXT factor ZFC3H1.

The nuclear RNA exosome targets many cryptic transcripts. Having established a PAXT-like phenotype of ZC3H3 and RBM26/RBM27 depletions by analyzing annotated transcripts, we next tested whether a similar relationship would be observed for unannotated transcripts. To this end, we focused on PROMPTs, the majority of which are NEXT-sensitive, but a subset of which are targeted by PAXT (Figure 4C, (12), and unpublished data). As above, we grouped these transcripts into NEXT (blue curves)- and PAXT (red curves)-sensitive classes and again found that ZC3H3 and RBM26/RBM27 depletions largely affected ZFC3H1-sensitive RNAs (Figure 4C). All together, we conclude that ZC3H3 and RBM26/RBM27 depletions impact PAXT but not NEXT targets.

## DISCUSSION

A considerable fraction of RNAPII-derived transcripts is degraded in mammalian nuclei by the RNA exosome (1). Short cryptic transcripts appear to be rapidly targeted, primarily by the NEXT exosome adaptor complex, whereas longer and more processed RNAs generally have extended nuclear residence times and are targeted by the PAXT connection both for their nuclear retention and their ultimate exosomal decay (12–14,20–22). It is believed that PAXT-mediated processes take place in competition with RNA nuclear export (14,40,41), although the underlying molecular principles still need to be fully delineated. Additionally, bearing in mind the large variety of targeted transcripts, it appeared plausible that additional protein factors contribute to nuclear exosome mediated RNA metabolism. One such auxiliary factor is PABPN1, which associates sub-stoichiometrically and in an RNA-dependent fashion with the core PAXT components MTR4 and ZFC3H1 (12). Inspired by these considerations, we chose two proteomic approaches aimed at identifying additional components functionally related to nuclear exosome function. Our work establishes ZC3H3 and RBM26/RBM27 as new factors involved in the turnover of pA^+^ RNA in mammalian nuclei.

Taking advantage of our earlier observation that the known PAXT components ZFC3H1 and PABPN1 accumulate with pA^+^ RNA in nuclear foci upon exosome depletion (14), we compared the nuclear pA^+^ RNA-bound proteomes between control and exosome compromised cells and found the ZnF protein ZC3H3 to be enriched upon exosome depletion (Figure 1C). In an alternative approach, aiming to discover new possible PAXT-related factors associating with MTR4 upon NEXT complex depletion, ZC3H3, PABPN1 and additionally the RNA-binding protein RBM27 were all enriched (Figure 3A). The functional relevance, in a PAXT context, of these candidate factors, ZC3H3, RBM27 and its paralog RBM26, was confirmed by the specific upregulation of PAXT targets upon their depletion (Figure 4). The fact that neither RBM26, RBM27 nor PABPN1 was enriched in the nuclear pA^+^ bound proteome of RRP40-depleted cells presumably reflects that these proteins also engage abundantly with RNAs independent of their PAXT linkage, i.e. all three factors are roughly ∼10- to 20-fold more abundant than ZFC3H1 in HeLa cells (42). This in turn underscores the necessity for diverse strategies to identify such proteins.

Characterization of the *S. pombe* MTREC complex identified proteins that bind to both Mtl1 (MTR4 ortholog) and Red1 (ZFC3H1 ortholog) (25–27). Interestingly, of these Red5 (ZC3H3 ortholog), Rmn1 (RBM26/RBM27 ortholog) and Pab2 (PABPN1 ortholog) were suggested to belong to the same MTREC submodule (25). Moreover, *Drosophila* dPABP2 (PABPN1 ortholog) and Swm (RBM27 ortholog) were among the most abundant proteins identified in a Drosophila dZC3H3 IP-MS study (43). It is therefore conceivable that ZC3H3, RBM26/RBM27 and PABPN1 constitute a mammalian counterpart of such a conserved composite. Between the Rmn1 homologs, RBM27 appears to be the principal PAXT component, with RBM26 functioning in a partially redundant manner, which was revealed only in the absence of RBM27 (Figure 3B and C). However, whether MTR4, ZFC3H1, ZC3H3, RBM26/27 and PAPBN1 provide the full complement of the PAXT connection, a holo-PAXT, is not clear. At least for some substrates, the pA polymerase gamma (PAPOLG) protein appears to play an important role (12,20–22), and consistently the *S. pombe* homolog, Pla1, was also found associated with MTREC (25–27,44) and has previously been implicated in the turnover of *S. pombe* transcripts by the RNA exosome (44). We note here that PAPOLG appears to be a strong interactor of ZFC3H1 (12,23), the full significance of which remains to be determined.

The MTR4-ZFC3H1 dimer constitutes the backbone of the PAXT connection. This was previously established by the high specific yields of MTR4 and ZFC3H1 in the reciprocal ZFC3H1 and MTR4 IPs, respectively (12,13). It is also supported by data from *S. pombe* where Mtl1 and Red1 form a central core of the MTREC complex (25–27). Finally, the link between MTR4 and PABPN1 fully depends on the presence of ZFC3H1 (12). However, while there is little doubt that the MTR4-ZFC3H1 dimer is central for PAXT activity, results presented in this paper imply that it is not the limiting factor. This is because, despite the higher affinity of MTR4 for ZFC3H1 than for the other PAXT components, our individual depletion of ZFC3H1, ZC3H3, RBM26/27 and PAPBN1 all yielded comparable abundance increases of PAXT substrates (Figures 2B and 4A) (12). Thus, these factors appear to play functionally equivalent roles in RNA decay. Moreover, ZCCHC8 depletion had no impact on the MTR4–ZFC3H1 interaction, while levels of ZC3H3, RBM27 and PABPN1 all increased in this condition (Figure 3A). Since the copy numbers of ZCCHC8 and ZFC3H1 in HeLa cells are comparable (∼30 000 molecules/cell, (42)), whereas ZC3H3 is less abundant (∼6000 molecules/cell), an interpretation of the latter result is to suggest a dynamic competition between the MTR4 to ZCCHC8-RBM7 (NEXT) and MTR4 to ZFC3H1-ZC3H3-RBM26/27-PABPN1 (holo-PAXT) assemblies, while the stable MTR4–ZFC3H1 dimer is less subject to protein exchange. This, in turn, would imply, that the MTR4–ZFC3H1 dimer is not sufficient to trigger RNA decay, which raises two new questions: (i) what is then the role of the presumably RNA-decay inactive MTR4–ZFC3H1 dimer and (ii) what is then the rate-limiting step for PAXT-mediated RNA turnover?

In addressing the first question, it might be relevant that ZFC3H1, in addition to its RNA decay affiliation, has been suggested to be central for the nuclear retention of pA^+^ RNA species and preventing their untimely association with cytoplasmic ribosomes (13,14). This ZFC3H1 activity might occur while the ultimate fate (nuclear export or decay) of pA^+^-RNPs is still being negotiated and therefore be kinetically separated from final assembly of holo-PAXT and the ensuing transcript decay (see below). It is also a possibility that the MTR4–ZFC3H1 dimer constitutes a form of ‘storage’ for one of the two components. It was recently shown that the NRDE2 protein can form a dimer with MTR4 and it was suggested that this provides negative regulation of MTR4 (45). An important research area for the future will therefore be to investigate the dynamic relationships of the different MTR4 and ZFC3H1 assemblies and how these manifest RNA production/destruction decisions.

Concerning the rate-limiting step for PAXT-mediated RNA decay, it is perhaps relevant to consider the low abundance of ZC3H3, which is at least 5-fold less than the remaining PAXT components described here (42). Consistent with a fundamental role in pA^+^ RNA turnover, ZC3H3 depletion triggers the formation of ZFC3H1-dependent nuclear pA^+^ RNA foci (Figure 2C) also observed upon exosome inactivation (Figure 1A) (14). Moreover, ZC3H3 interacts with ZFC3H1 in an RNA-independent manner (Figure 2A), placing the protein in close proximity with MTR4 and eventually the nuclear exosome. Hence, it is conceivable that ZC3H3’s engagement with the MTR4–ZFC3H1 dimer is a vital step in assembling the holo-PAXT connection and committing it to RNA decay. Whereas the ZFC3H1–ZC3H3 interaction is RNase insensitive, PABPN1 engages with ZFC3H1 and ZC3H3 through RNA mediated interactions (Figure 2A) (12). We originally proposed that the pA^+^ RNA-binding activity of PABPN1 provides substrate specificity (12). However, the RNA-binding capabilities of the new PAXT proteins RBM26 and RBM27 as well as the potential contributions of RNA-binding activities of the ZnF domains of ZFC3H1 and ZC3H3 provide an array of additional RNA-binding possibilities. It would therefore be tempting to speculate that a variety of individual RNA-binding segments serve in the recognition of different substrate classes, which together with PABPN1 allows for targeting of a wide variety of polyadenylated transcripts. However, this notion is not directly supported by our data, which instead demonstrate that individual factor depletions affect a similar set of transcripts (Figures 2B, 3C and 4). Therefore, an alternative possibility is that final recruitment of the RNA exosome requires the co-operative binding of several factors to elicit RNA decay. PABPN1, RBM26 and RBM27 are abundant RNA-binding proteins, which likely contact RNAs independently of PAXT function. Hence, perhaps binding by several factors at the same time is required to elicit decay. Given a competition between nuclear RNA decay and export, step-wise assembly of the full holo-PAXT connection would give the RNA export system some leeway to act before destruction of the transcript is triggered. This scenario is also compatible with a proposed model of pA-tails, constituting ‘molecular timers’ for nuclear RNA degradation and where the nuclear residence time of a given polyadenylated RNA determines its half-life in the nucleus (8,46). A major mechanistic determinant for this model could therefore be the kinetics by which the full PAXT connection assembles. Details of such assembly and the exact factor dependencies still need to be resolved, which will ultimately be a high throughput endeavor as different substrates may behave differently.

## Supplementary Material

gkz1238_Supplemental_Files
