# Supplementary material for: The human ZC3H3 and RBM26/27 proteins are critical for PAXT-mediated nuclear RNA decay
Source: Nucleic Acids Res. 2020 Jan 17;48(5):2518–30. doi: 10.1093/nar/gkz1238 (PMC7049725; doi:10.1093/nar/gkz1238)
Supplement: gkz1238_Supplemental_Files [file gkz1238_supplemental_files.zip › Table S4.docx]

| type | # | siZCCHC8/siEGFP | siRRP40/siEGFP | siZFC3H1/siEGFP | siZC3H3/siEGFP | siRBM2627/siEGFP |
| --- | --- | --- | --- | --- | --- | --- |
| misc_RNA | 3602 | 28 | 78 | 66 | 169 | 199 |
| lnc_RNA | 6746 | 158 | 496 | 303 | 364 | 547 |
| mRNA | 25852 | 239 | 555 | 382 | 795 | 1087 |
| snoRNA | 149 | 1 | 4 | 0 | 0 | 1 |
| antisense_RNA | 10 | 0 | 1 | 1 | 2 | 2 |
| snRNA | 26 | 3 | 5 | 2 | 0 | 0 |
