# Supplementary figures and images for: The human ZC3H3 and RBM26/27 proteins are critical for PAXT-mediated nuclear RNA decay
Source: Nucleic Acids Res. 2020 Jan 17;48(5):2518–30. doi: 10.1093/nar/gkz1238 (PMC7049725; doi:10.1093/nar/gkz1238)

Figure S1

A

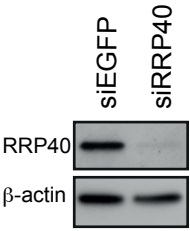

B

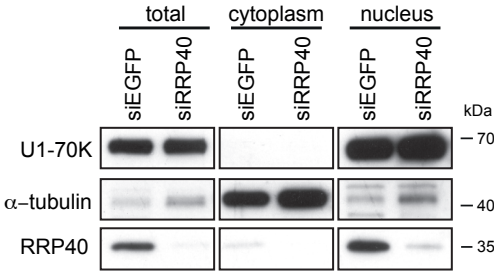

C

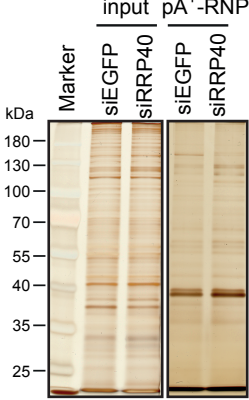

Supplement: gkz1238_Supplemental_Files [file gkz1238_supplemental_files.zip › Sup_Figure1.pdf]

Figure S2

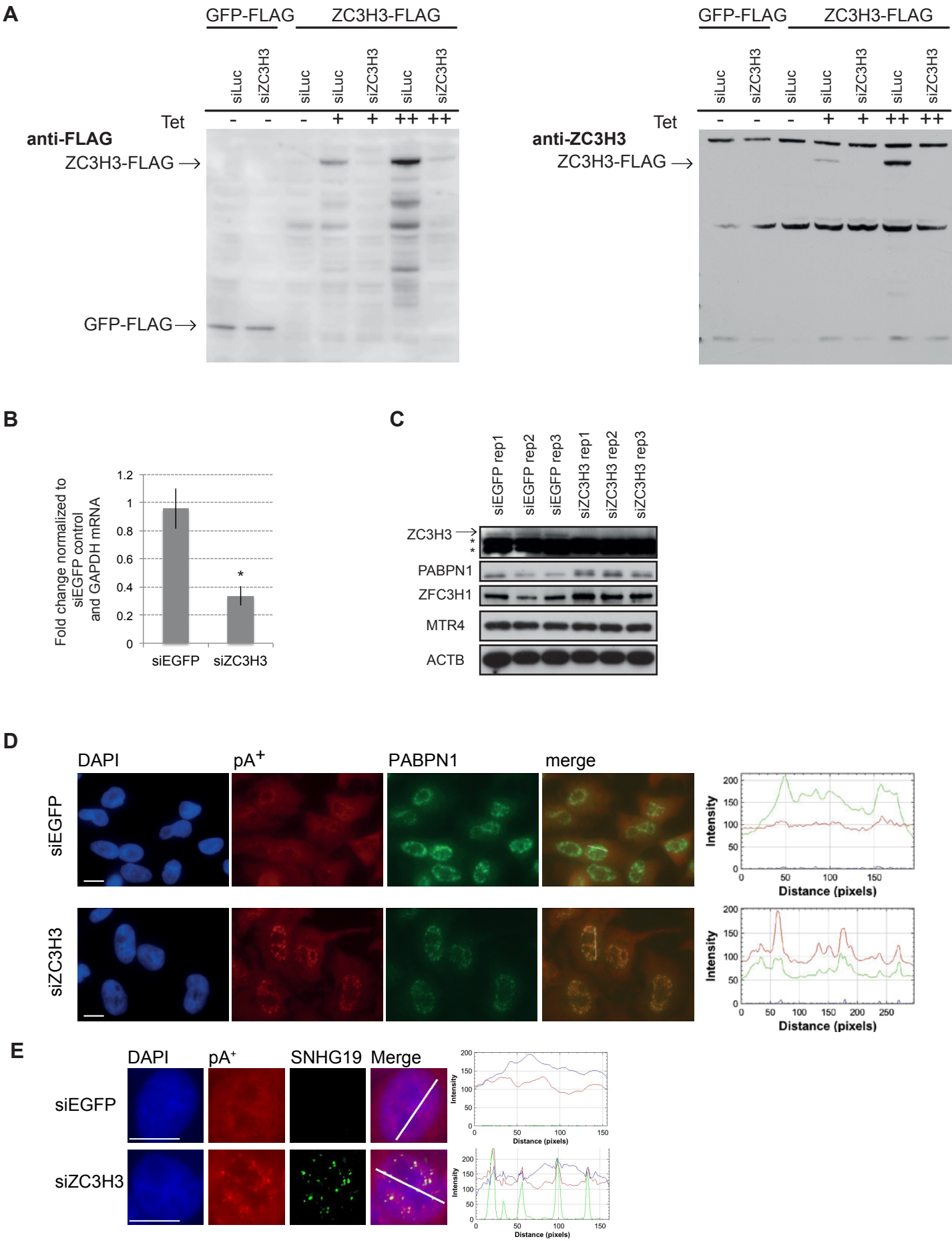

Supplement: gkz1238_Supplemental_Files [file gkz1238_supplemental_files.zip › Sup_Figure2_revised.pdf]

Figure S3

A

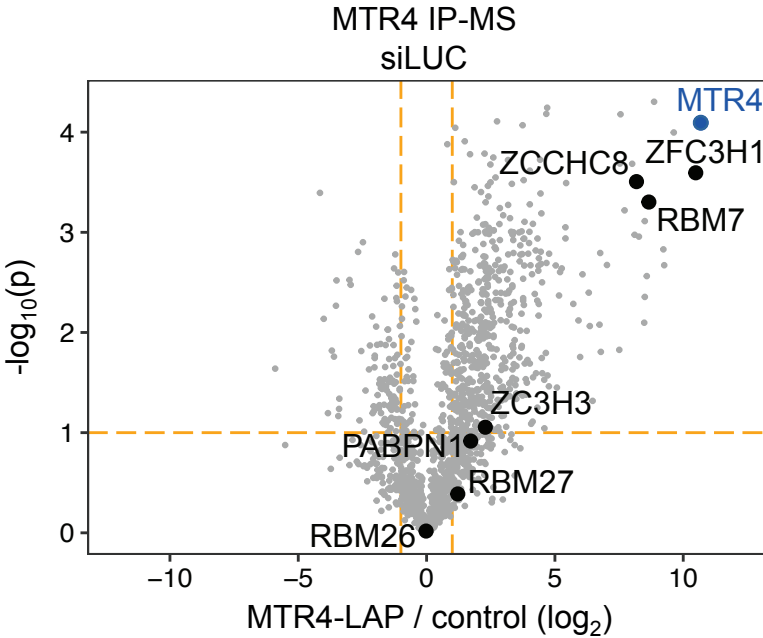

B

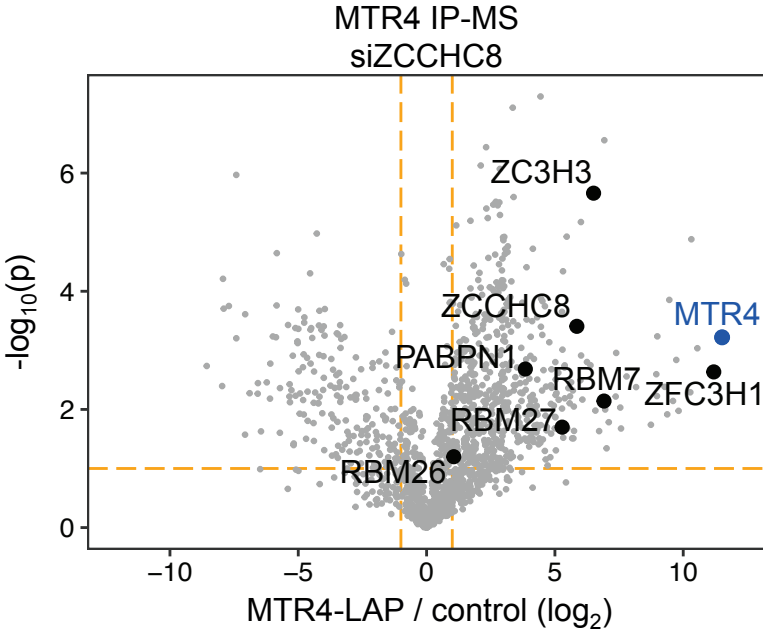

C

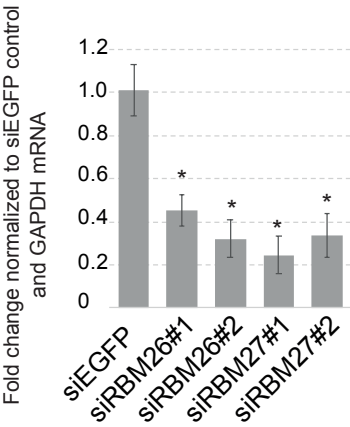

D

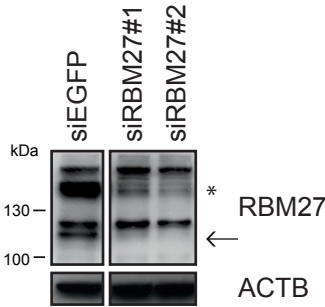

Supplement: gkz1238_Supplemental_Files [file gkz1238_supplemental_files.zip › Sup_Figure3.pdf]

Figure S4

A

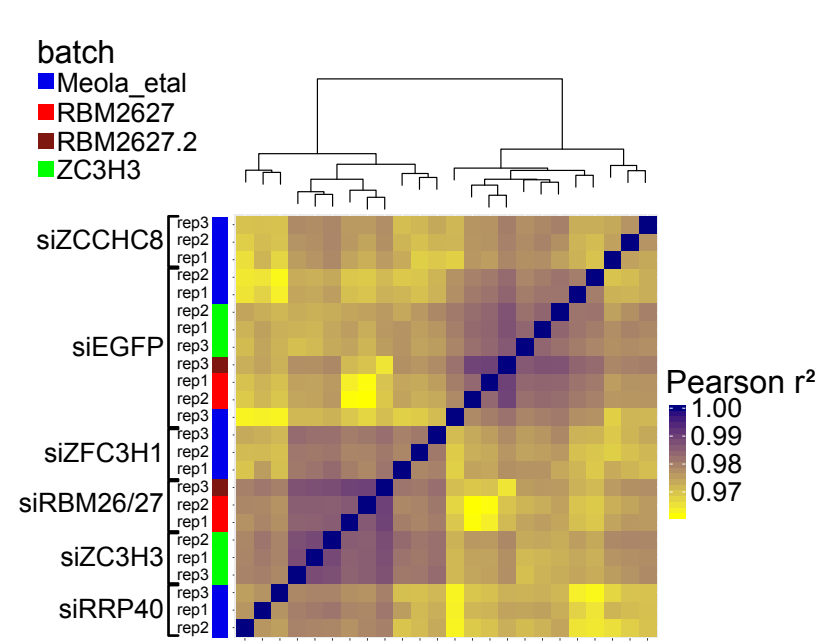

B

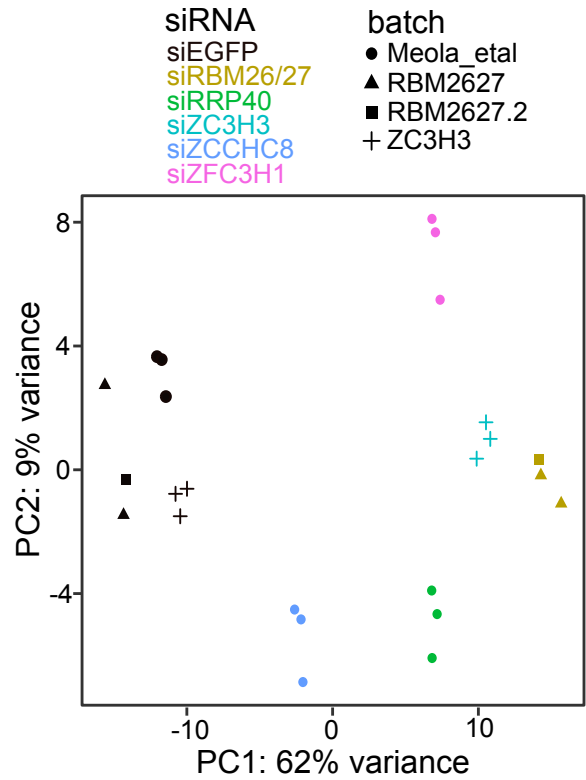

Supplement: gkz1238_Supplemental_Files [file gkz1238_supplemental_files.zip › Sup_Figure4.pdf]
